# Micro/Nanoscale Structured Superhydrophilic and Underwater Superoleophobic Hybrid-Coated Mesh for High-Efficiency Oil/Water Separation

**DOI:** 10.3390/polym12061378

**Published:** 2020-06-19

**Authors:** Teng Yuan, Jian Yin, Yingling Liu, Weiping Tu, Zhuohong Yang

**Affiliations:** 1 Guangdong Laboratory of Lingnan Modern Agricultural Science and Technology, Key Laboratory for Biobased Materials and Energy of Ministry of Education, College of Materials and Energy, South China Agricultural University, Guangzhou 510642, China; yinjian@stu.scau.edu.cn (J.Y.); liuyingling@stu.scau.edu.cn (Y.L.); 2 Guangdong Provincial Key Lab of Green Chemical Product Technology, School of Chemistry and Chemical Engineering, South China University of Technology, Guangzhou 510640, China; cewptu@scut.edu.cn; 3 Department of Applied Physics, The Hong Kong Polytechnic University, Hung Hom, Kowloon, Hong Kong 999077, China

**Keywords:** superhydrophilic, underwater superoleophobic, micro-nanoscale binary rough structure, coated mesh, oil–water separation

## Abstract

A novel micro/nanoscale rough structured superhydrophilic hybrid-coated mesh that shows underwater superoleophobic behavior is fabricated by spray casting or dipping nanoparticle–polymer suspensions on stainless steel mesh substrates. Water droplets can spread over the mesh completely; meanwhile, oil droplets can roll off the mesh at low tilt angles without any penetration. Besides overcoming the oil-fouling problem of many superhydrophilic coatings, this superhydrophilic and underwater superoleophobic mesh can be used to separate oil and water. The simple method used here to prepare the organic–inorganic hybrid coatings successfully produced controllable micro-nano binary roughness and also achieved a rough topography of micro-nano binary structure by controlling the content of inorganic particles. The mechanism of oil–water separation by the superhydrophilic and superoleophobic membrane is rationalized by considering capillary mechanics. Tetraethyl orathosilicate (TEOS) as a base was used to prepare the nano-SiO_2_ solution as a nano-dopant through a sol-gel process, while polyvinyl alcohol (PVA) was used as the film binder and glutaraldehyde as the cross-linking agent; the mixture was dip-coated on the surface of 300-mesh stainless steel mesh to form superhydrophilic and underwater superoleophobic film. Properties of nano-SiO_2_ represented by infrared spectroscopy and surface topography of the film observed under scanning electron microscope (SEM) indicated that the film surface had a coarse micro–nano binary structure; the effect of nano-SiO_2_ doping amount on the film’s surface topography and the effect of such surface topography on hydrophilicity of the film were studied; contact angle of water on such surface was tested as 0° by the surface contact angle tester and spread quickly; the underwater contact angle to oil was 158°, showing superhydrophilic and underwater superoleophobic properties. The effect of the dosing amount of cross-linking agent to the waterproof swelling property and the permeate flux of the film were studied; the oil–water separation effect of the film to oil–water suspension and oil–water emulsion was studied too, and in both cases the separation efficiency reached 99%, which finally reduced the oil content to be lower than 50 mg/L. The effect of filtration times to permeate flux was studied, and it was found that the more hydrophilic the film was, the stronger the stain resistance would be, and the permeate flux would gradually decrease along with the increase of filtration times.

## 1. Introduction

Research on superhydrophobic [1,2], superoleophobic [3,4], superamphiphobic [4,5,6,7] and superoleophilic [8,9,10] surfaces has developed rapidly in recent years [11,12,13,14,15,16,17,18,19,20]. Meanwhile, an increasing number of applications for such surfaces, including self-cleaning [21], anti-fingerprint coatings [22], anti-fog coatings [23], microdroplet transfer technology [24] and oil–water separation [25,26,27,28,29,30], have been investigated. Superhydrophilic surfaces have been drawing extensive attention since Fujishima found that nanoscale TiO_2_ possesses superhydrophilic properties when exposed to light and recovers its hydrophobic nature in the dark with cyclic reversibility [31]. However, how to prepare stable superhydrophilic surfaces under natural conditions has seldom been reported. The wettability of a liquid on a solid surface is mainly driven by surface chemistry, which is determined by surface geometry [32,33,34,35]. To fabricate superoleophobic or superhydrophobic surfaces, materials with a low surface energy and micro–nano binary rough structures are required [36,37,38]. Thus, superoleophobic or superhydrophobic surfaces are generally fabricated by constructing a rough structure on a low-energy surface or grafting low-energy materials from a rough surface [39,40,41]. From the perspective of pure surface chemistry, a superlyophilic surface requires the surface tension of the solid and liquid to be close, while superoleophobic or superhydrophobic surfaces require the surface tension of the solid to be less than a quarter that of the liquid material [42]. Because the surface tension of oils (usually 20–40 mN/m) and solid materials is usually low, most solids are potentially oleophilic and hydrophobic [43,44,45,46]. According to recently reported data, the surface tension of a fluoropolymer of 10.4 mN/m is the lowest among solids, while that of water (72.8 mN/m) is the highest among liquids [47]. Thus, if we prepare a superoleophilic surface, it is highly possible that it is simultaneously hydrophobic or superhydrophobic [48]. Therefore, it is relatively easy to prepare superhydrophobic and superoleophilic membranes, as they only need the corresponding rough structure to be included once the requirements of surface tension are met.

Many superhydrophobic and superoleophilic membranes and their applications have been reported [49,50,51]. However, because of their potential oleophilicity, these membranes possess a high adhesion force to oil; that is, it is very easy for oil to adhere to the surface or pores of these membranes [52,53,54]. This can make cleaning the surface difficult and cause irreversible blockage of membrane pores, greatly limiting the lifetime and scope of application of such membrane materials. On the basis of the original research, the opposite idea, that is, the so-called “water-removing” method, has been used to prepare superhydrophilic and superoleophobic membranes [55,56,57]. The contact angle of superhydrophilic and superoleophobic membranes against oil is greater than 150° in air and water [58]. Meanwhile, these membranes possess an extremely low adhesion force, and the rolling angle is only 2–3°, which effectively prevents the adhesion of oil droplets [59]. Such materials can act as efficient anti-pollution separation membranes. However, the surface tension of the membrane needs to be lower than that of oil to generate a superoleophobic surface. As a result, the corresponding surface tension of the film is far lower than that of water, so the surface is also a superhydrophobic interface. This means that superhydrophilic and superoleophobic properties are a contradiction between two opposites [60,61,62].

Hydrophilic polymer hydrogels possess good hydrophilicity and are water sensitive, as well as demonstrating good adhesion and film-forming properties [63]. However, they readily dissolve in water, so superhydrophilic membranes prepared from hydrophilic polymer hydrogels exhibit poor water resistance, and it is very difficult to assemble an ideal micro-nanoscale binary rough structure using a hydrogel-coated wire mesh. Hydrophilic polymer emulsions show good hydrophilicity, water absorption and water retention, but are not fully soluble in water. Achieving a homogeneous dispersion of such emulsions in water relies on the emulsification of the emulsifier [64]. Hydrophilic polymer emulsions possess good adhesion and film-forming properties, and the resulting films have excellent water resistance. In addition, nano-SiO_2_ particles possess excellent hydrophilicity because of the large number of hydroxyl groups present on their surfaces [65,66,67]. Hydrophilic nano-SiO_2_ particles can be dispersed in water in the form of nanoparticles, so it is easy to assemble micro–nano structures on the surface of organic polymer membranes. However, because of the poor film-forming properties of inorganic nanoparticles, it is difficult to form a film of nano-SiO_2_ particles alone.

In this paper, a simple dipping and drying method and organic–inorganic hybrid were used to prepare superhydrophilic oil–water separation membranes exhibiting excellent performance. A hydrophilic polymer emulsion and nano-SiO_2_ particles were used as film-forming adhesives and inorganic hybrid components, respectively. Polyvinyl alcohol (PVA) was added as a hydrophilic polymer and polymeric emulsifier, and stainless steel wire mesh was used as a substrate.

## 2. Materials and Methods

### 2.1. Materials and Instruments

Tetraethyl orathosilicate (TEOS, chemical pure) was purchased from Dongguan Xinyu High Polymer Materials Co., Ltd. (Dongguan, China). Polyvinyl alcohol (PVA) (PVA 1799, degree of polymerization = 1700, degree of alcoholysis = 99%, industrial grade) was purchased from China Petrochemical Corporation. Methyl methacrylate (MMA, chemically pure), butyl acrylate (BA, chemically pure), styrene (St, chemically pure), methacrylic acid (MAA, chemically pure), alcohol (chemically pure), ammonia hydroxide (chemically pure), acetone (chemically pure) and sodium dodecyl benzene sulfonate (SDBS, chemically pure) were purchased from Tianjin Kemiou Chemical Reagent Co., Ltd. (Tianjin, China). Glutaraldehyde (chemically pure) was purchased from DOW Chemical Company. Dimethylbenzene (industrial grade) was purchased from Guangzhou Baiyun Chemical Plant (Guangzhou, China). Stainless steel mesh (300-mesh) was purchased from Guangzhou Pingxiang Screen Factory (Guangzhou, China). Distilled water used was from the laboratory. All chemicals were used as received without further purification.

Electronic scales AY-120 were from SHIMADZU (Kyoto, Japan); an electro-thermostatic blast oven DHG-9140A was from Shanghai Yiheng Technology Co., Ltd. (Shanghai, China); a digital thermostatic water bath HH-4 was from Changzhou Guohua Electric Appliance Co., Ltd.(Guangzhou, China); an infrared spectrometer Perkin-Elmer1730 was from Perkin Elmer (Waltham, US); a micro surface contact meter Dataphysics OCA40 was from Dataphysics (Stuttgart, Germany); and a scanning electron microscope S-3700N was from Hitachi (Tokyo, Japan).

### 2.2. Preparation of a Nano-Silica Sol Using the Sol–Gel Technique

**Scheme 1:**
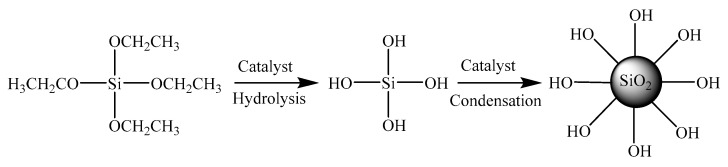
Preparation of nano SiO_2_ by ethyl silicate via hydrolytic condensation.

Tetraethoxysilane (50 g), anhydrous ethanol (100 g) and deionized water (20 g) were added to a 500-mL four-necked flask and stirred in a water bath at a constant temperature of 35 °C (Scheme 1). After uniform mixing, 25% ammonia (0.5 g) in deionized water (30 g) was added over around 30 min with a current pump. The resulting solution was incubated for 4 h to give silica nanoparticles with a size of about 20 nm.

### 2.3. Synthesis of Polymer Latex Microspheres

**Scheme 2:**
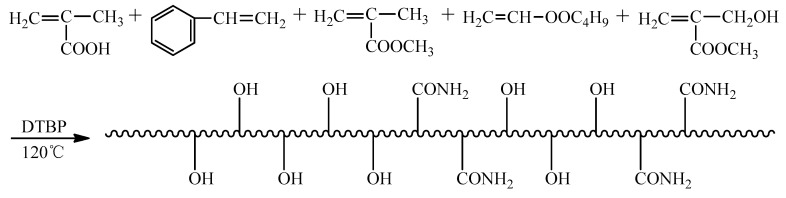
Preparation of the hydroxyl acrylic resin via radical polymerization.

Methyl methacrylate (MMA), butyl acrylate (BA), styrene (St) and methacrylic acid (MAA) were used as monomers, sodium dodecyl benzene sulfonate (SDBS) was used as an emulsifier, sodium persulfate (SPS) was used as an initiator and NaHCO_3_ was used as a buffer. An acrylic emulsion was synthesized using the radical emulsion polymerization method (Scheme 2). Deionized water (50 g) and anionic emulsifier SDBS (0.0126 g) were placed in a 250 mL four-necked flask equipped with a stirring paddle, thermometer, condenser pipe and nitrogen gas inlet valve. The mixture was stirred and heated gradually to 85 °C, and then MAA (0.225 g), St (2.2 g) and SPS (0.2 g) were added sequentially. The mixture was reacted for 1 h, and then kept warm for subsequent use. Next, deionized water (120 g) was placed in a 1000 mL round bottom four-necked flask equipped with a stirring paddle, thermometer, condenser pipe and nitrogen gas inlet valve. Monomer mixture (prepared by mixing St (60 g) and MAA (1.2 g)) and 1-dodecanethiol (3 g) were added sequentially. The stirred mixture was heated to 85 °C. Then the above seed emulsion was added dropwise over 1 h to form a core emulsion of encapsulated hydrocarbon. The separate phases served as the polymerization core in the next step. Half of an aqueous initiator solution prepared by mixing deionized water (120 g) with SPS (1.056 g) was added over 75 min to the above core polymer latex microsphere solution, and then the remainder was added dropwise over 225 min simultaneously with a mixture of deionized water (50 g), anionic surfactant DNS-458 (3 g), St (100 g), DVB (21.6 g) and MAA (2 g). The reaction mixture was stirred at 85 °C for 1 h.

### 2.4. Preparation of PVA Aqueous Solution

A 1000 mL beaker was thoroughly cleaned, dried and weighed, and the mass was marked as m_1_; then, 760 g deionized water was added into the beaker in a 97 °C thermostatic water bath, and the mixture was stirred until the water temperature reached 97 °C; later, under stirring, 40 g PVA was slowly added into the beaker until the PVA was thoroughly dissolved. The water solution was put aside until it cooled down naturally and the bubbles disappeared completely; the flask was weighted again, and the mass was then marked as m_2_; the deionized water was added again until the mass reached m_1_ + 800 − m_2_; the mixture was then stirred for subsequent use.

### 2.5. Preparation of Superhydrophilic Membranes

**Figure 1:**
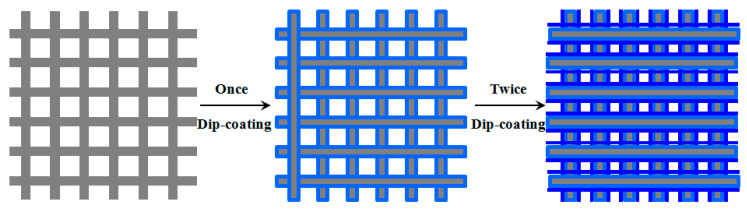
Schematic of preparation of superhydrophilic and underwater superoleophobic composite membrane via a two-step coating method.

The 300-mesh stainless steel mesh was sequentially cleaned ultrasonically with water, acetone or ethanol, and water and then dried at room temperature. A stirred solution of the hydrophilic polymer water-sensitive agent and crosslinking agent dissolved in water was mixed with the nano-sol mixed ultrasonically to give a hybrid solution. The mesh was immersed in the hybrid solution for 5 min and then removed vertically (Figure 1). Alternatively, the hybrid solution was sprayed or spin coated directly on the mesh. Then the coated mesh was dried in a vacuum oven at 100–200 °C to give superhydrophilic and underwater superoleophobic membranes suitable for oil–water separation. A second coating was applied by repeating the above steps.

### 2.6. Characterization

Fourier transform infrared (FT-IR) spectra were obtained on an FT-IR spectrometer (MAGNA-IR750, Nicolet, Madison, US) using KBr pellets. The SiO_2_ nanoparticles were dried at 120 °C before preparing the KBr pellet. Scanning electron microscope (SEM) images were obtained on a SEM (S-3700N, Hitachi, Tokyo, Japan) operating at 15.0 kV. Contact angles were measured on a contact-angle system (OCA40, Dataphysics, Stuttgart, Germany) at ambient temperature. Water or diesel oil droplets (about 4.0 mL) were carefully added onto the coating films. Contact angle values were obtained by measuring contact angles at five different positions of the same sample.

## 3. Results and Discussion

### 3.1. Infrared Characterization

**Figure 2:**
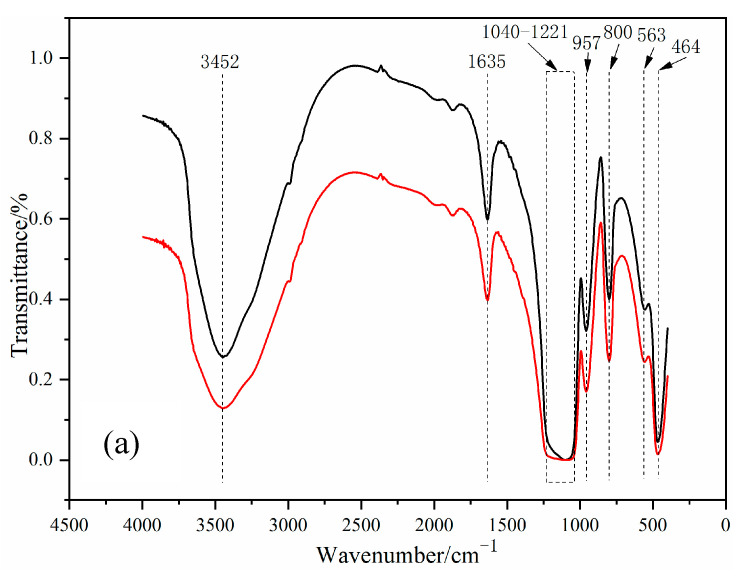

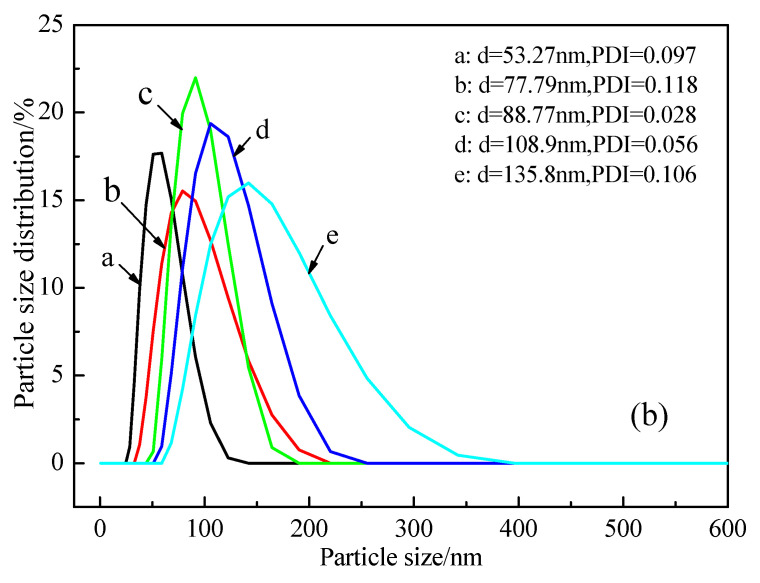
IR spectra (**a**) and particle size distribution (**b**) of the SiO_2_ nanoparticles.

Figure 2 presents FT-IR spectra and particle size distribution of the prepared SiO_2_ nanoparticles with a hydroxyl-rich surface. The FT-IR spectra had a strong absorption peak at 3452 cm⁻^1^ consistent with the absorption peak of hydroxyl –OH, one at 957 cm⁻^1^ from the stretching vibration of Si–OH silanol, a very strong, broad absorption at 1040–1221 cm⁻^1^ from Si–O stretching vibration and an Si–O–Si (fourth ring body) stretching vibration. These data all indicated that tetraethoxysilane hydrolysis and condensation generated SiO_2_ nanoparticles with hydroxyl-rich surfaces.

### 3.2. SEM Characterization

**Figure 3:**
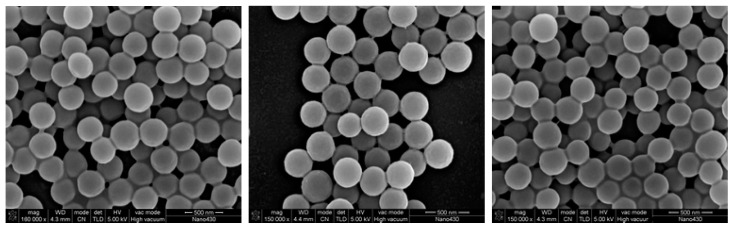
SEM images of different sizes of acrylic polymer nano-latex particles.

Polymer nano-latex microspheres with uniform size and good monodispersity were prepared by in situ polymerization through solvent-less encapsulation. As shown in Figure 3, the prepared latex particles had a regular structure, with good monodispersity, a smooth, hard surface and particle diameter of 275–480 nm.

**Figure 4:**
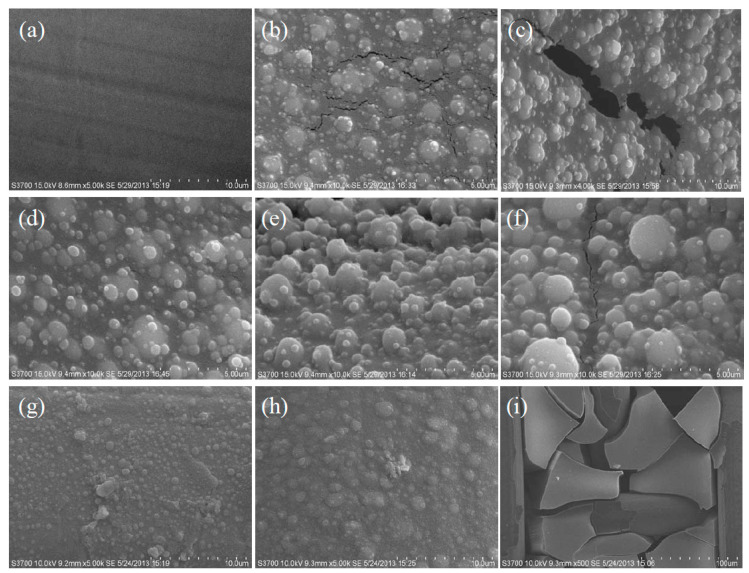
SEM images of hybrid-coated mesh films prepared from a stainless steel mesh with an average pore diameter of about 50 mm. The content of SiO_2_ is (**a**) 33%, (**b**) 40%, (**c**) 50%, (**d**) 60%, (**e**) 66%, (**f**) 75%, (**g**) 80%, (**h**) 90% and (**i**) 100%.

Membranes containing different contents of SiO_2_ nanoparticles in organic polymer were prepared. SEM was used to observe the surface morphology of the membranes, as shown in Figure 4. Figure 3 shows that when the SiO_2_ content was less than 40 wt %, the coating on the membrane surface was very smooth with almost no projections. When the content of SiO_2_ nanoparticles was greater than 40 wt %, the coating surface was evenly distributed with a large number of micro- and nanosized projections. When the content of SiO_2_ nanoparticles was 40–75 wt %, the mesh was covered with microsized particles of 1–3 μm and nanoparticles of 100–300 nm.

When the content of SiO_2_ nanoparticles was greater than 80 wt %, few microscale protrusions were observed on the membrane surface. Instead, there was a more uniform distribution of nanoscale projections, so the surface looked smooth. When the membranes were prepared using only nano-SiO_2_, the membrane surface was smooth but broke into small pieces because of the poor film-forming properties of nano-SiO_2_. Although the hydrophilicity of nano-SiO_2_ was excellent, its water resistance was poor, so the nano-SiO_2_ could be easily washed off the stainless steel mesh.

The membranes formed in this study suggested that the nano-SiO_2_ hybridized with the hydrophilic polymer emulsions. The hydrophilic polymer emulsion acted as an emulsifier, effectively preventing the reunion of nano-SiO_2_. When the content of SiO_2_ was too low, the surface of the membrane was smooth because of excess polymer covering the nanoparticles. However, when the SiO_2_ content was 40–75 wt %, nanoparticles were uniformly present in the emulsion in the form of nano-emulsion pellets that formed a micro-nano projection structure. When the SiO_2_ content exceeded 80 wt %, because of the relatively small content of the emulsion, there was better emulsification, resulting in uniform dispersion of nano-SiO_2_. As a result, only nanoscale projections were formed. When the membranes were prepared using just nano-SiO_2_, because of the absence of emulsifier, the nanoparticles aggregated during film formation, thus resulting in a smooth flat surface. From the above analysis, we concluded that when the SiO_2_ content was 60–75 wt %, a highly structured monolayer with micro-nano structure was obtained on the substrate.

**Figure 5:**
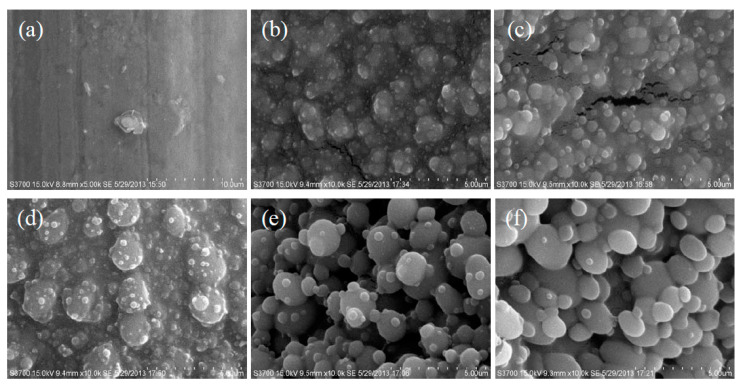
SEM images of hybrid-coated mesh films after secondary coating. The SiO_2_ content is (**a**) 33%, (**b**) 40%, (**c**) 50%, (**d**) 60%, (**e**) 66% and (**f**) 75%.

The hybrid-coated mesh film prepared with SiO_2_ contents of 33, 40, 50, 60, 66 and 75 wt % were dipped again in the same casting solution to form a second coating. The surface morphology of these double-coated membranes was observed by SEM, as shown in Figure 5.

Figure 5 reveals that when the SiO_2_ content was less than 40 wt %, the surface of the film was still relatively smooth after the secondary coating. When the SiO_2_ content was 40–75 wt %, micro-nanoscale structured craters were clearly observed on the surface of the films after secondary coating and the size of the particles was almost unchanged. However, when the content of SiO_2_ was greater than 60 wt %, micro-nanoscale structured craters with a multilevel distribution were clearly observed on the surface of the film after secondary coating. These films contained more pores in their surface after secondary coating, so the surface roughness of the film was increased, improving their hydrophilicity. When the SiO_2_ content was 66 wt %, the most structured, layered micro-nano structures could be obtained on a substrate after two coatings, resulting in the highest hydrophilicity and oleophobic behavior underwater, giving the highest oil–water separation efficiency of the investigated membranes. When the substrates were coated three or more times, the pores tended to be plugged, so although a multi-level micro–nano structure could be obtained, the rate of water infiltration was low.

### 3.3. Contact Angle Characterization

**Figure 6:**
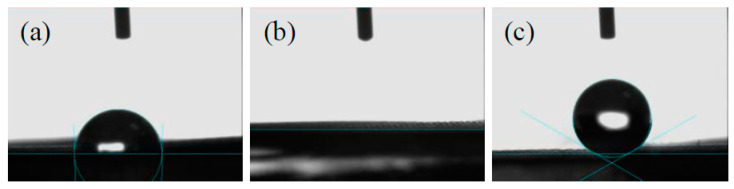
Contact angle of water on (**a**) the clean stainless steel mesh surface (91.3°), (**b**) the superhydrophilic membrane (0°), (**c**) contact angle of oil on the superhydrophilic membrane under water (158°).

**Figure 7:**
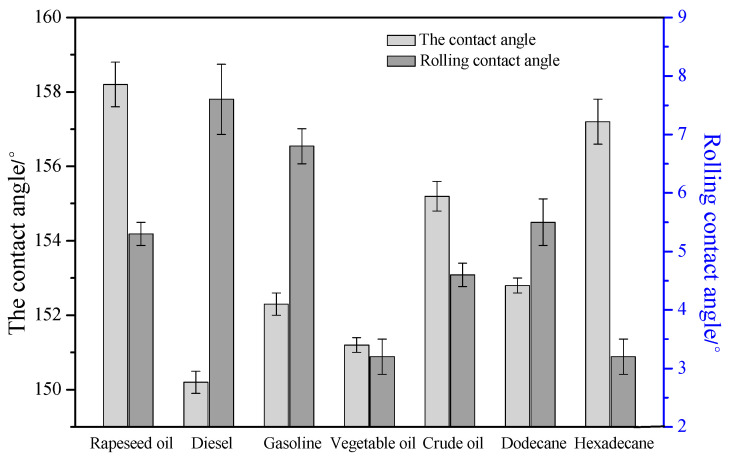
The contact angle and rolling contact angle of various types of oils.

Contact angles (CAs) of the blank clean mesh and the superhydrophilic mesh, respectively, to the 3 μmL water in the air were 91.7° ± 0.3° and 0° as measured by the surface tension tester (OCA); and contact angle of the film surface to oil was 158.4 ± 0.3° underwater (Figure 6 and Figure 7), indicating the superhydrophilic and underwater superoleophobic properties of the film.

(1) $$\cos\theta_{OW}=\frac{\gamma_{OA}\cos\theta_{O}^{}-\gamma_{WA}\cos\theta_{W}}{\gamma_{OW}}$$

Superhydrophilic and underwater superoleophobic films demonstrate the superhydrophilic and superoleophobic properties in the air, that is, their contact angles to water and oil are both 0°. Water has the highest surface tension (about 72.8 mN/m) among all liquids. The surface tension of dimethyl benzene is 28.7 mN/m and the interfacial tension of the same to water is 47.9 mN/m. The theoretical contact angle of dimethyl benzene underwater to the film surface is obtained (158.9°) by substituting the aforesaid values into the following formula, which is in coincidence with that measured by the experiment. This is because the hydrophilic polymer and doped inorganic hydrophilic nanoparticles on the hybrid mesh, as well as the micro-nano coarse structure formed by the same, enhanced the connection between water and film surfaces and thus produced a water film among the micro–nano structures on the film surface and consequently reduced the contact area between the dimethyl benzene and the film and increased the contact angle, so that underwater, the superoleophobic property was acquired.

### 3.4. Application Results

**Figure 8:**
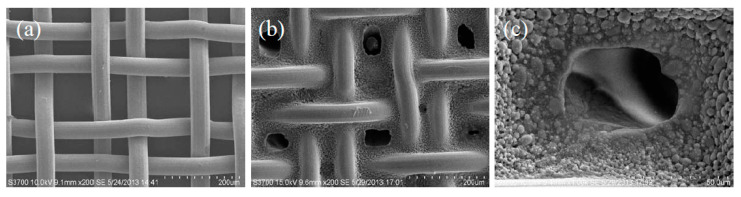
SEM images of the hybrid-coated mesh film prepared using stainless steel mesh with an average pore diameter of about 50 mm. (**a**) Large-area view of the stainless steel mesh, (**b**) large-area view of the hybrid -coated mesh film, and (**c**) enlarged view of the hybrid-coated mesh film.

The clean 300-mesh stainless steel mesh and prepared superhydrophilic mesh were observed by SEM; the results are shown in Figure 8. The 300-mesh stainless steel screen had pores with a diameter of about 50 μm, wire diameter of about 40 μm, and a smooth surface. In contrast, the surface of the superhydrophilic membrane was very rough with micro- and nanostructure protrusions and micro- and nanopores distributed in the grid. In addition, micro-nanoscale structured craters could be clearly observed around the membrane pores. The membrane can be used to separate oil and water mixtures because of its superhydrophilic properties. When a mixture of oil and water contacts the surface of the membrane, gradually the water moves below the oil because it has a higher density than the oil. The superhydrophilic properties of the membrane causes water to rapidly spread over the surface of the film and occupy the surface, so a solid–liquid–liquid phase boundary is formed on the micro–nano structured rough surface. Under the action of gravity and capillary forces, a steady stream of water can penetrate the membrane and move through it. As the water occupies the roughened surface, a hydration layer is formed on the surface of the film, and because of the incompatibility of oil and water, the surface of the film becomes oleophobic at this point. According to the Wenzel equation, for a lyophobic surface, the greater the degree of surface roughness, the larger the contact angle of the liquid on the surface. The surface of the film at this time becomes underwater superoleophobic, and the oil receives upward capillary resistance. Because of this capillary force, the oil cannot penetrate the surface of the film, provided the gravity generated by the oil is not greater than the capillary resistance.

**Figure 9:**
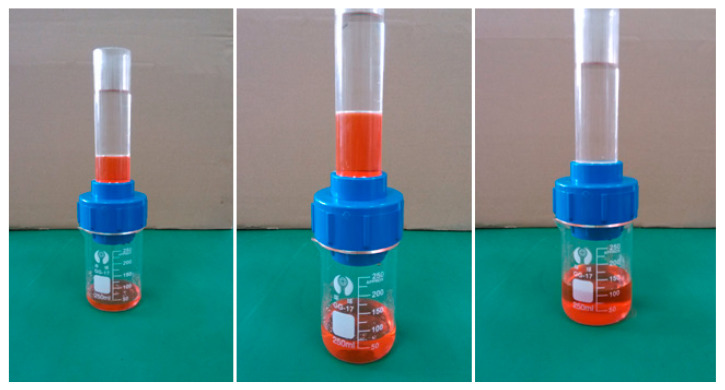
Oil–water separation experiment performed using the hybrid-coated mesh.

To examine the separation capability of the superhydrophilic oil–water separation membranes, water was dyed with red ink and mixed with dodecane in a volume ratio of 1:1 and then stirred to form a uniform mixture. The oil–water mixtures were poured into a device containing the membrane, as shown in Figure 9. The hydrophilic polymers swelled after absorbing water, and a steady stream of red water permeated into the lower beaker. Meanwhile, the dodecane was blocked above the membrane. Ultimately, there was no red residue left on the membrane, so efficient separation of an oil–water mixture was achieved.

**Figure 10:**
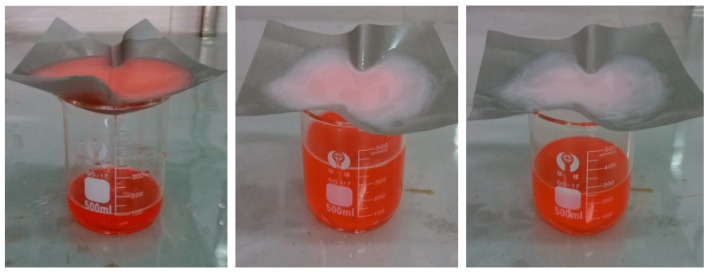
Renderings of membrane separation of oil and water emulsion.

**Figure 11:**
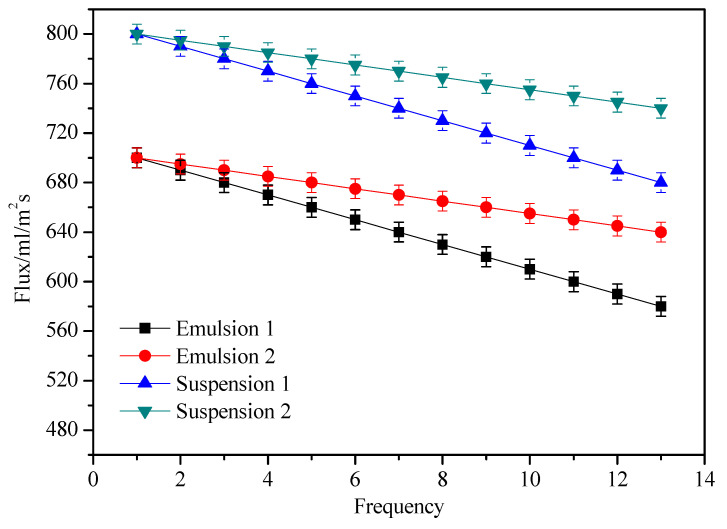
Effect of the number of membrane filtration on flux.

The water was dyed red with ink to prepare the dimethyl benzene–water solution with a mass concentration of 3 g/L; the mixture was shaken with ultrasonic waves to set free and disperse the oil for separation experiment so that the effect of the oil phase on separation could be studied (Figure 9). See Figure 10 for final results and Figure 11 for effects of filtration times on film permeate flux. It can be seen that the red water could quickly and thoroughly permeate through the film, with no red substances left on the film, but the colorless dimethyl benzene was left on the film.

The dimethyl benzene–water solution was prepared with mass concentration of 3 g/L, and the emulsifier SDBS was added into the solution, and the mixture was magnetically stirred until the oil reached the O/W emulsion state for further separation experiments to observe the effects of the oil phase on separation performance of the film (Figure 10). It can be seen that the red water could quickly and thoroughly permeate through the film and only the white dimethyl benzene emulsion was left on the film, since the ink was aqueous and could fully dissolve in water and permeate, leaving only the nearly white dimethyl benzene. Thus, this film showed a good separation effect on the oil–water emulsion.

Presence of oil phase in the solution hardly influenced the separation effects of the film. When the oil phase was present as a semi-emulsion, the oil content in water decreased from the initial 3 g/L to below 50 mg/L after 1h separation; when the oil phase existed as an emulsion, the oil content in water also decreased from the initial 3 g/L to below 50 mg/L after 1 h separation.

It can be seen from Figure 11 that the permeate flux to separate the oil–water suspension was greater than that to separate the oil–water emulsion. This because demulsification is required when separating oil–water emulsions, and the size of O/W micelle particles in an oil–water emulsion is bigger than that of free water in the oil–water suspension, and thus the O/W micelle particles can be filtered quickly. The permeate flux of film will slowly decrease along with the increase of filtration times regardless of what kind of oil–water mixture is being separated. The reason for this is that during the constant filtration process, oil drops will contact the film surface constantly to cause pollution. However, through comparison of the two kinds of film with different doping amounts of nanoparticles, it is noted that the bigger the doping amount, the slower the decrease of permeate flux and vice versa. Because the best doping amount is within the range of 40–75%, the increase of doping amount of nano particle will give the film better hydrophilicity. Thus, a conclusion can be drawn: the better hydrophilicity the film has, the stronger the stain resistance the film has. With better hydrophilicity, the stronger the adhesion strength between film and water and thus a water layer is attached firmly on the film surface and thus effectively prevents the pollution of oil drops on the film surface.

### 3.5. Separation Mechanism

The separation mechanism of the membrane can be explained from the viewpoint of capillary mechanics. In general, a thin tube with an inner diameter of less than 1 mm is referred to as a capillary. The phenomenon where liquid rises along the edge of a pore and then penetrates or descends when in contact with a thin pore is called capillarity. It is generally believed that the experimental intrusion pressure is the pressure that is generated by the height of a liquid column of oil, while the theoretical intrusion pressure is the capillary pressure. However, in fact, a third force is also believed to exist during this process, namely a liquid bridge force. Capillary action occurs when the adhesion between the liquid and solid (the wall) is greater than the cohesion of the liquid itself. When a certain amount of liquid that can wet the solid surface with short distance is present, a system called a liquid bridge can form, driven by the adhesion force between the two solid surfaces and liquid.

The liquid bridge has two main functional characteristics, namely transportation and connection. The transportation characteristics manifest as heat convection and mass transfer of the liquid. The connection characteristics manifest as the drag force of the liquid acting on the two solid surfaces to form a continuous liquid membrane. Regardless of the liquid mass transfer or heat transfer convection, or the drag force of the liquid acting on the two solid surfaces, the root of these properties is the surface tension of the liquid. A minor reason why a liquid bridge force is generated is the surface tension of the curved surface of the liquid, whereas major reasons are the adhesion between the liquid and solid and the cohesion of liquid. When the liquid makes contact with a wettable solid, adhesion attraction is generated between the liquid and surface of the solid, and cohesion attraction exists in the liquid, which causes the liquid to have a surface drag effect on the solid in the liquid bridge. Experiments have shown that when the liquid is water and the solid surface is hydrophilic, adhesive strength is greater than cohesive strength. As the water adheres to the superhydrophilic membrane, a liquid bridge is formed, which is equivalent to forming an oleophobic layer in a capillary, and effectively prevents the penetration of oil droplets through the membrane.

## 4. Conclusions

In conclusion, superhydrophilic membranes were prepared by a simple dipping and drying method using organic–inorganic hybrid materials. When the proportion of inorganic nanoparticles was 40–70 wt %, rough micro–nano composite structures could be formed on the surface of the mesh, endowing the mesh surface with superhydrophilic properties. Micro–nano multilevel structures were constructed on the mesh surface after two coatings, which could further increase the surface area and surface roughness of the film, resulting in better hydrophilic properties. The developed superhydrophilic membranes were used for oil–water separation, giving a separation efficiency of more than 99 wt %.
